# Supplementary material for: Unlocking the functional potential of polyploid yeasts
Source: Nat Commun. 2022 May 11;13:2580. doi: 10.1038/s41467-022-30221-x (PMC9095626; doi:10.1038/s41467-022-30221-x)
Supplement: Supplementary file 3 — Description of Additional Supplementary Files [file 41467_2022_30221_MOESM3_ESM.pdf]

Description of Additional Supplementary Files

File Name: Supplementary Data 1
Description: Strains used in the study

File Name: Supplementary Data 2
Description: High impact mutations OS1364

File Name: Supplementary Data 3
Description: High impact mutations OS1431

File Name: Supplementary Data 4
Description: Primers and oligos used in this study

File Name: Supplementary Data 5
Description: RTG assay performed

File Name: Supplementary Data 6
Description: Stressor phenotyping of WT RTGs and control samples

File Name: Supplementary Data 7
Description: Main genes involved in fermentation used for the analysis

File Name: Supplementary Data 8
Description: Recombination detected in fermentation associated genes

File Name: Supplementary Data 9
Description: Lab scale wort fermentation results

File Name: Supplementary Data 10
Description: Lab scale wort fermentation results

File Name: Supplementary Data 11
Description: Sugar Consumption, Post fermentation viability and Aroma analysis

File Name: Supplementary Data 12
Description: Stat reciprocal non reciprocal AF shifts in M-D WT and ndt80 mutant RTG sectors
