# Supplementary material for: Unlocking the functional potential of polyploid yeasts
Source: Nat Commun. 2022 May 11;13:2580. doi: 10.1038/s41467-022-30221-x (PMC9095626; doi:10.1038/s41467-022-30221-x)
Supplement: Supplementary file 16 — Reporting Summary [file 41467_2022_30221_MOESM16_ESM.pdf]

nature portfolio

Corresponding author(s): Gianni Liti
Simone Mozzachiodi

Last updated by author(s): 06/04/2022

# Reporting Summary

Nature Portfolio wishes to improve the reproducibility of the work that we publish. This form provides structure for consistency and transparency in reporting. For further information on Nature Portfolio policies, see our Editorial Policies and the Editorial Policy Checklist.

## Statistics

For all statistical analyses, confirm that the following items are present in the figure legend, table legend, main text, or Methods section.

| n/a | Confirmed | |
|---|---|---|
| ☐ | ☒ | The exact sample size (*n*) for each experimental group/condition, given as a discrete number and unit of measurement |
| ☐ | ☒ | A statement on whether measurements were taken from distinct samples or whether the same sample was measured repeatedly |
| ☐ | ☒ | The statistical test(s) used AND whether they are one- or two-sided<br>*Only common tests should be described solely by name; describe more complex techniques in the Methods section.* |
| ☒ | ☐ | A description of all covariates tested |
| ☐ | ☒ | A description of any assumptions or corrections, such as tests of normality and adjustment for multiple comparisons |
| ☐ | ☒ | A full description of the statistical parameters including central tendency (e.g. means) or other basic estimates (e.g. regression coefficient) AND variation (e.g. standard deviation) or associated estimates of uncertainty (e.g. confidence intervals) |
| ☐ | ☒ | For null hypothesis testing, the test statistic (e.g. *F*, *t*, *r*) with confidence intervals, effect sizes, degrees of freedom and *P* value noted<br>*Give P values as exact values whenever suitable.* |
| ☒ | ☐ | For Bayesian analysis, information on the choice of priors and Markov chain Monte Carlo settings |
| ☒ | ☐ | For hierarchical and complex designs, identification of the appropriate level for tests and full reporting of outcomes |
| ☒ | ☐ | Estimates of effect sizes (e.g. Cohen's *d*, Pearson's *r*), indicating how they were calculated |

*Our web collection on statistics for biologists contains articles on many of the points above.*

## Software and code

Policy information about availability of computer code

| | |
|---|---|
| Data collection | The kinetic parameters of the growth phenotypes obtained in the 100 well plate phenotypic assay were extracted with GrowthCurver v 0.3.0 (https://cran.r-project.org/web/packages/growthcurver/vignettes/Growthcurver-vignette.html) |
| Data analysis | Sequencing data were analysed using the following softwares<br>and associated scripts are going to be deposited in a public repository:<br>- R 4.0.4 (2021-02-15)<br>- R 3.6.1 (2020-02-15)<br>- NUCmer - 4.0.0 beta<br>- LRSDAY -1.6.0<br>- flowCore - 1.52.1<br>- bwa - 0.7.17-r1198-dirty<br>- samtools - 1.11<br>- freebayes - 1.3.4<br>- bedtools - 2.29.2<br>- bcftools - 1.14-7<br>- Growthcurver - 0.3.0<br>- Ensembl Variant Effect Predictor (VEP) https://www.ensembl.org/info/docs/tools/vep/index.html<br>- https://github.com/SimoneMozzachiodi/UnlockingFunctionalPotentialofPolyploidYeasts |

For manuscripts utilizing custom algorithms or software that are central to the research but not yet described in published literature, software must be made available to editors and reviewers. We strongly encourage code deposition in a community repository (e.g. GitHub). See the Nature Portfolio guidelines for submitting code & software for further information.

# Data

Policy information about availability of data

All manuscripts must include a data availability statement. This statement should provide the following information, where applicable:

- Accession codes, unique identifiers, or web links for publicly available datasets
- A description of any restrictions on data availability
- For clinical datasets or third party data, please ensure that the statement adheres to our policy

The phenotype data are available within the supplementary data sets. The short reads sequences generated in this study are available at the SRA, NCBI, under the accession code PRJNA770168.

# Field-specific reporting

Please select the one below that is the best fit for your research. If you are not sure, read the appropriate sections before making your selection.

☐ Life sciences ☐ Behavioural & social sciences ☒ Ecological, evolutionary & environmental sciences

For a reference copy of the document with all sections, see nature.com/documents/nr-reporting-summary-flat.pdf

# Ecological, evolutionary & environmental sciences study design

All studies must disclose on these points even when the disclosure is negative.

| | |
|---|---|
| Study description | This study aimed to develop a framework in which the return to growth (RTG) protocol can be used to rapidly generate genetic recombination in sterile polyploid strains and unlock novel phenotypes. We used two unrelated sterile industrial strains that we characterised for meiotic progression and gamete viability, confirming their extreme sterility. Then, we selected 11 candidates RTG and 2 control samples for each genetic background by using a selective marker for which we followed the loss of heterozygosity (LOH) upon RTG. Then, we developed a selection system based on colony phenotype variability and selected a total of 48 GMO-free strains and 10 ndt80 mutants as control of the meiotic progression. Finally, we performed whole genome sequencing of the evolved RTG strains mentioned above and characterised them in environments mimicking industrial fermentations. The samples were subselected based on the phenotypic characterisation and for the 2L scale fermentation test, two GMO-free RTG strains for each parental strain were used. |
| Research sample | The RTG samples sequenced in this study were derived from two polypliod strains that had different genome content and ancestry. We decided to use two different polyploid strains to show that our framework can be exported to different genetic backgrounds. These two polyploid strains aim to be two case studies of polyploid industrial strains.<br>The parental strains where obtained from - Peter J, De Chiara M, et al. Genome evolution across 1,011 Saccharomyces cerevisiae isolates. Nature. 2018 Apr;556(7701):339-44. |
| Sampling strategy | The samples sequenced in this study were obtained from RTG plates where cells were plated at different time-points. The strains selected with the LYS2/URA3 system were taken from 5-FOA plates where we plated the meiotic culture after 14 hours of meiotic progression. We selected 11 RTG colonies from the 5-FOA plates o f each of the two parents to be sure to have enough cells that had engaged successfully in RTG.<br>RTG sectoring strains were obtained from the plating of the meiotic cells on media containing different concentrations of dextrose. Particularly, cells of the two sectors were collected from the same colony by collecting part of the different edges showing phenotypic variability, otherwise, when not specified, a whole colony was taken. In this case we aimed to take at least 10 independent RTG colonies. |
| Data collection | Sequencing was performed by the Institut Curie NGS platform. For each batch, the following "instrument/flow-cell/paired-end insert-size/library-preparatation kit" were used:<br>- D167 -> NovaSeq – SP flow cell – PE150 – Nextera XT<br>- D287 -> NovaSeq6000 – SP flow cell – PE150 – KAPA Hyper Prep<br>- D415 -> NovaSeq6000 – SP flow cell – PE150 – KAPA Hyper Prep<br>- D536 -> NovaSeq6000 – SP flow cell – PE150 – KAPA Hyper Prep |
| Timing and spatial scale | No field data collection was performed. |
| Data exclusions | No samples were excluded. |
| Reproducibility | For the estimation of LOH rate with the LYS2/URA3 system, 5 independent replicates were performed for each hybrid. Spore viability was estimated on 400 spores dissected across two different dissection experiments. The estimation of the sectoring phenotype was performed by counting the colony sectoring phenotype on the overall number of colonies in 8 technical replicates. The stressor phenotype performed on 100 well plate was done using 4 technical replicates for each strain. The flask scale fermentation was performed on 2 technical replicates. The fermentation experiment in the 2L vessel was performed in 3 independent technical replicates. The aroma profile and post-fermentation viability data where obtained analysing the 3 technical replicates used for th 2L fermentation. All attempts at replication were successful. |

| | |
|---|---|
| Randomization | Samples were not divided into groups. |
| Blinding | Blinding was not relevant for the study. E.g., in order to analyse the sequencing data, short-reads from evolved samples had to be genotyped and compared for the allelic variation with the respective parental strain. |

Did the study involve field work? ☐ Yes ☒ No

# Reporting for specific materials, systems and methods

We require information from authors about some types of materials, experimental systems and methods used in many studies. Here, indicate whether each material, system or method listed is relevant to your study. If you are not sure if a list item applies to your research, read the appropriate section before selecting a response.

## Materials & experimental systems

| n/a | Involved in the study |
|---|---|
| ☒ | ☐ Antibodies |
| ☒ | ☐ Eukaryotic cell lines |
| ☒ | ☐ Palaeontology and archaeology |
| ☒ | ☐ Animals and other organisms |
| ☒ | ☐ Human research participants |
| ☒ | ☐ Clinical data |
| ☒ | ☐ Dual use research of concern |

## Methods

| n/a | Involved in the study |
|---|---|
| ☒ | ☐ ChIP-seq |
| ☐ | ☒ Flow cytometry |
| ☒ | ☐ MRI-based neuroimaging |

# Flow Cytometry

## Plots

Confirm that:

☐ The axis labels state the marker and fluorochrome used (e.g. CD4-FITC).

☒ The axis scales are clearly visible. Include numbers along axes only for bottom left plot of group (a 'group' is an analysis of identical markers).

☐ All plots are contour plots with outliers or pseudocolor plots.

☒ A numerical value for number of cells or percentage (with statistics) is provided.

## Methodology

| | |
|---|---|
| Sample preparation | Cells were first pulled out from glycerol stocks on solid YPD media and kept overnight at 30 °C. The following day a small number of cells was taken from a patch with a pipette tip and resuspended in liquid YPD media and incubated overnight at 30 °C. The next day, cells where washed once with water and fixed overnight in 70% cold ethanol at 4 °C. To conclude, cells where washed once with phosphate buffer saline (PBS), and 100 µL of each sample were resuspended in 900µL of staining solution (15µM PI, 100µg/mL RNase A, 0.1% v/v Triton-X, in PBS) and incubated for 3 hours at 37°C in the dark. |
| Instrument | FACS-Calibur flow cytometer |
| Software | The data were analyzed using FlowCore and FlowViz to divide the cell population in different bins according to the fluorescence detected with a FL2.A filter and then plotted with ggplot2 (R version 3.6.1) in order to detect the peak of G1 or G2 cells characteristics of the ploidy of the strain. |
| Cell population abundance | Cells were re-suspended after a overnight culture to reach a final concentration of 1000-500 cells/µL in each sample used. |
| Gating strategy | The distribution of fluorescence induced by staining with Propidium iodide (PI) was analysed to find the two main density peaks. These peaks correspond to the two cell populations, respectively in G1 and G2 phases and are represented as a density plot in supplementary figure 6D. The peak with the smaller values, which corresponds to the cell in G1, was used to determine the ploidy compared to a reference strain with known ploidy without gating. |

☐ Tick this box to confirm that a figure exemplifying the gating strategy is provided in the Supplementary Information.
